# Gene editing in dermatology: Harnessing CRISPR for the treatment of cutaneous disease

**DOI:** 10.12688/f1000research.23185.2

**Published:** 2020-10-07

**Authors:** Catherine Baker, Matthew S. Hayden

**Affiliations:** 1 Geisel School of Medicine at Dartmouth, Hanover, NH, 03755, USA; 2 Section of Dermatology, Surgery, Dartmouth-Hitchcock Medical Center, Lebanon, New Hampshire, 03766, USA

**Keywords:** CRISPR, dermatology, gene editing, genodermatoses, viruses, cutaneous disease

## Abstract

The discovery of the Clustered Regularly Interspaced Short Palindromic Repeats (CRISPR) system has revolutionized gene editing research. Through the repurposing of programmable RNA-guided CRISPR-associated (Cas) nucleases, CRISPR-based genome editing systems allow for the precise modification of specific sites in the human genome and inspire novel approaches for the study and treatment of inherited and acquired human diseases. Here, we review how CRISPR technologies have stimulated key advances in dermatologic research.  We discuss the role of CRISPR in genome editing for cutaneous disease and highlight studies on the use of CRISPR-Cas technologies for genodermatoses, cutaneous viruses and bacteria, and melanoma. Additionally, we examine key limitations of current CRISPR technologies, including the challenges these limitations pose for the widespread therapeutic application of CRISPR-based therapeutics.

## Introduction

Gene editing technologies have been transformative in biological research and show immense potential for the study and treatment of inherited and acquired human diseases ^1^. The Clustered Regularly Interspaced Short Palindromic Repeats (CRISPR) system uses programmable RNA-guided CRISPR-associated (Cas) nucleases to change, remove, or add genetic material to specific locations within the genome ^2^. In comparison with other site-specific nucleases, such as zinc finger nucleases (ZFNs), meganucleases (MNs), and transcription activator-like effector nucleases (TALENs), CRISPR-Cas nucleases are easier to design and implement through the simple manipulation of a guide RNA sequence.

Dermatologic conditions hold particular appeal as targets for CRISPR-Cas therapeutics. There are several well-described, monogenic inherited skin disorders, such as the epidermal blistering disorders, that are considered ideal candidates for genome editing therapeutics. In addition, the skin is an easily accessible organ that allows for extraction and *in vitro* culture of target cells as well as direct localized administration of CRISPR-Cas therapeutics through topical, grafting or injection methods ^3^. Lastly, for the same reason, the visibility of skin allows for simpler monitoring of the genetically edited cutaneous tissues for both efficacy and potential deleterious effects ^3^.

Ongoing research efforts are exploring a variety of CRISPR-Cas approaches to the development of new CRISPR-Cas therapeutics for dermatology. Though most experiments have focused on *ex vivo* manipulation of diseased primary cell lines, researchers are increasingly developing *in vivo* and *ex vivo* techniques with translational potential. We propose that, for a variety of reasons, dermatology is likely to continue to be at the center of the development and clinical application of CRISPR-Cas therapeutics. For example, one of the first human trials involving CRISPR-Cas9 is geared toward treating refractory melanoma, among other neoplasms ^4^. Therefore, in this review we will focus on the current research and potential future applications of therapeutic CRISPR-Cas nucleases in dermatology.

## Mechanisms of genome engineering with CRISPR-Cas

**Figure 1.**
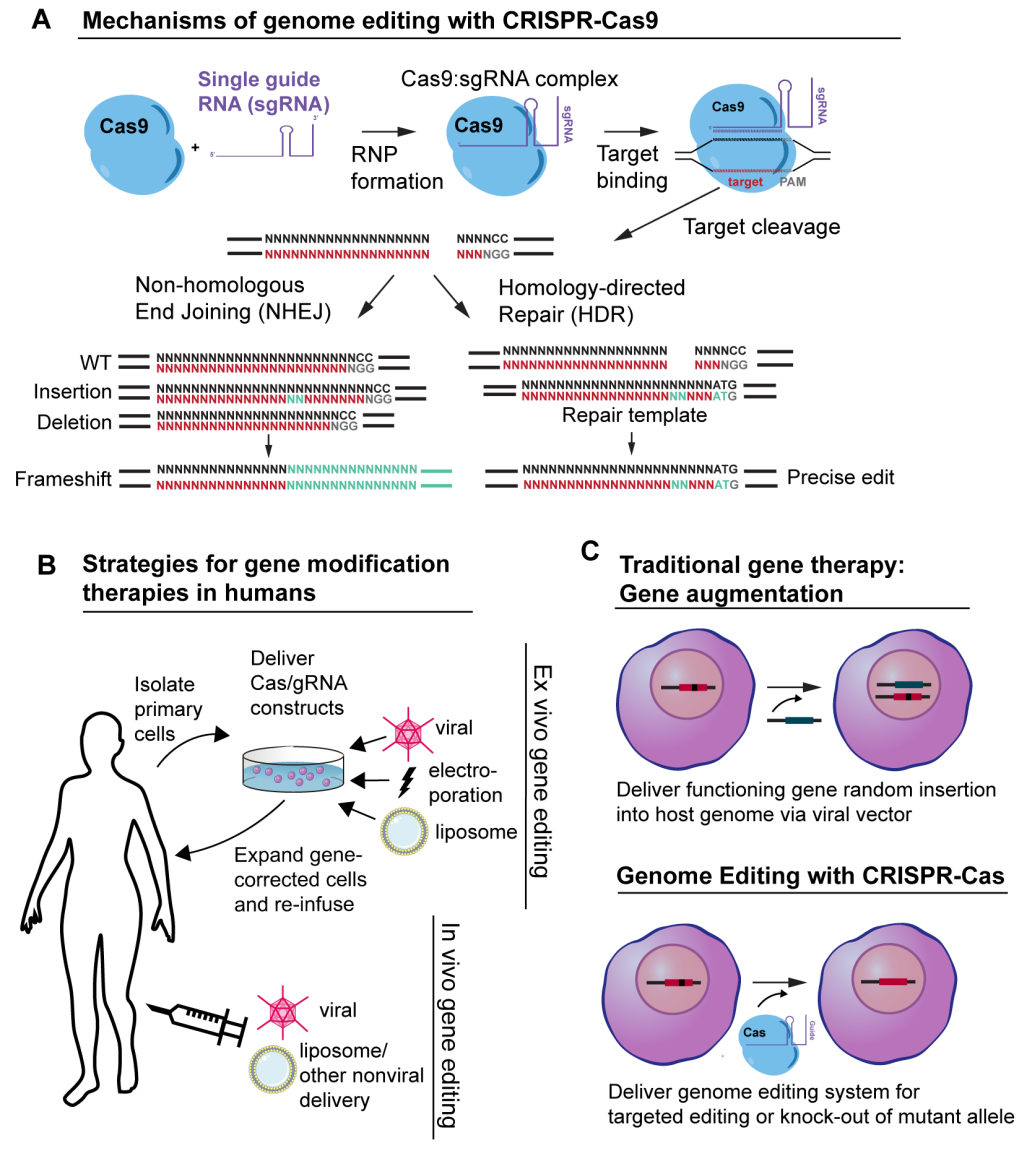
CRISPR-Cas9 gene editing strategies. ( **A**) Mechanism of CRISPR-Cas9 genome editing. Cas9 nuclease complexes with a single guide RNA (sgRNA) to form a ribonucleoprotein (RNP). The sgRNA guides Cas9 to create a double strand break (DSB) three to four base pairs proximal to an “NGG” PAM sequence. After creating a DSB, dsDNA can be repaired by either non-homologous end joining (NHEJ) or, when a homologous dsDNA donor template is available, homology-directed repair (HDR). ( **B**) Strategies for gene modification therapies in humans. *Ex vivo* gene editing strategies involve the extraction and manipulation of patient-derived cells *in vitro* in cell culture. Gene-corrected cells are expanded in culture and are subsequently re-infused or grafted onto the patient. *In vivo* gene editing involves the direct delivery of CRISPR-Cas DNA, RNA, and/or protein via viral or nonviral means. ( **C**) Traditional gene therapy versus genome editing with CRISPR-Cas technology. Traditional gene therapy involves the addition of a functioning gene to replace a mutant allele. The replacement gene is usually inserted randomly into the host genome via a viral vector. In contrast, genome editing with CRISPR-Cas involves the direct, site-specific editing of the host genome.

There are several types of CRISPR-Cas systems (I-III), and numerous subtypes, that have been identified in bacteria and archaea, but the type II CRISPR-Cas9 system is the best studied, particularly in terms of its application to dermatology therapeutics ^5^. The type II CRISPR system provides bacteria with a mechanism of immunologic memory and defense against foreign DNA ^6^. Using CRISPR, bacteria incorporate short sequences of exogenous DNA from invading pathogens, for example from bacteriophages or viruses that infect bacteria, into their own genome. When transcribed from the bacterial host genome, these sequences are processed into CRISPR RNAs (crRNAs) that complex with a *trans*-activating crRNA (tracrRNA). The crRNA/tracRNA duplex directs Cas9 to cleave target double-stranded DNA that is complementary to the 20-nucleotide guide sequence within the crRNA, creating a site-specific double strand break (DSB). In the laboratory, a single 80 to 100-nucleotide RNA transcript synthesized in the form of a single guide RNA (sgRNA) can mimic the structure and function of the crRNA/tracrRNA duplex ( Figure 1) ^2^. The simplicity and multiplexing capacity of CRISPR-Cas9 nuclease activity is based on the easy-to-design sgRNA or crRNA, whose RNA sequences can be modified to direct the Cas nuclease to target different sequences in the dsDNA genome.

In eukaryotic cells, following the formation of a site-specific DSB by Cas9, one of two cellular repair processes can occur: non-homologous end joining (NHEJ) or homology-directed repair (HDR) ( Figure 1) ^7^. NHEJ is an error-prone process that can result in mutations or nucleotide insertions and deletions (indels), interrupting the sequence of a target gene. In contrast, HDR is a high-fidelity DNA repair strategy whereby the DSB is repaired using homologous DNA as a template. HDR can be facilitated by co-administration of homologous donor DNA with the Cas nuclease. This donor sequence can be used as a synthetic template for the cell to copy when repairing the Cas-induced DSB. HDR can be used to direct the repair of a mutated gene, albeit with lower efficiency than NHEJ ^8^. Approaches are still being developed to improved HDR efficiency, which currently is found to vary widely depending on the target and targeting approach ^9, 10^.

To date, most genome engineering strategies for dermatological disease have involved the *ex vivo* editing of patient-derived primary cells ( Figure 1) ^11^. To perform *ex vivo* editing, patient cells are isolated and genetically modified *in vitro*, potentially for subsequent autologous transplantation. This strategy allows for the clonal selection, characterization, and expansion of genetically modified cells prior to use for engraftment in the affected organ or tissue. *Ex vivo* approaches facilitate targeting and delivery of the CRISPR-Cas therapeutic and, by allowing for enrichment of modified cells, reduce the requirement for highly efficient and specific CRISPR-Cas editing constructs ^12^. However, cell expansion in culture can lead to unwanted cellular differentiation, particularly in induced pluripotent stem cells (iPSCs) ^13^. In addition, cell-based transplantations can be technologically challenging, especially for non-hematopoietic cells. In contrast to *ex vivo* gene manipulation, *in vivo* gene editing involves the direct modification of somatic cells *in situ* ( Figure 1). Using CRISPR-Cas constructs, *in vivo* gene editing is achieved through systemic or local administration of packaged CRISPR-Cas components (protein, DNA, and/or RNA) into the body to induce gene editing outcomes in specific organs or cells. *In vivo* editing requires the development of effective targeting strategies to generate cell-specific changes with minimal off-target effects and precludes comprehensive characterization of all edited cells. Safe *in vivo* gene editing techniques could have utility for a wide range of systemic and localized diseases, but many hurdles and concerns remain to be addressed.

### Genodermatoses

Most genodermatoses are monogenic in nature and therefore serve as an attractive disease model for gene therapy ^14^. Because there are no widely available effective treatments for these disorders, current therapies are focused primarily on symptom management. Early success in the use of gene therapy for the treatment of the monogenic inherited epidermolysis bullosa (EB) disorders provided particular promise for the development of curative therapies for genodermatoses. In 2006, a patient suffering from nonlethal junctional EB (JEB) underwent successful long-term skin transplantation with epidermal sheets made from gene-corrected autologous keratinocytes ^15, 16^. These keratinocytes were corrected using a retroviral vector encoding the beta 3 chain of laminin-332, compensating for the mutated version of the gene in this patient. Such successes laid the groundwork for further research in gene replacement and gene editing strategies for cutaneous disorders. Unlike classically defined gene therapy, which involves the random insertion of one or more exogenous genes into cells to replace the function of a missing or mutated gene, gene editing involves the direct, site-specific manipulation of the genome using targeted nucleases such as ZFNs, TALENs, and CRISPR-Cas enzymes ( Figure 1) ^17^. The therapeutic potential of direct genome manipulation is immense. Researchers have leveraged CRISPR-Cas constructs to develop diverse treatment strategies for genodermatoses including the targeted addition of genes to specific genomic sites, the correction of disease-causing point mutations, and the removal of disease-causing genes or genomic sequences. Such strategies allow for corrective gene editing and the targeted ‘knockout’ of mutant alleles in dominant negative disorders. It is also hoped that targeted gene editing strategies will reduce the risks associated with random insertion of exogenous transgenes.

***EB***. Of the genodermatoses, the inherited EB disorders have been the most extensively studied as potential candidates for gene editing therapy ^3^. The EB disorders are a diverse group of inherited blistering diseases that affect the skin and, in some subtypes, mucous membranes and other organs ^18^. They are caused by mutations in over 20 different genes that code for different proteins expressed at the cutaneous basement membrane zone ^19^.

Two EB subtypes—EB Simplex (EBS) and dominant dystrophic EB (DDEB)—are caused by dominant negative mutations that cannot be corrected with traditional additive gene therapies. Thus, these disorders are particularly well-suited for treatment with CRISPR-Cas genome editing, which allows for the direct modification of the dominant, disease-causing allele. EBS is caused by dominant negative missense mutations in either the keratin 14 ( *KRT14*) or keratin 5 ( *KRT5*) genes that code for intermediate filaments (IFs) expressed in the basal layer of the epidermis ^20, 21^. Kocher *et al.* ^22^ used CRISPR-Cas9-induced HDR to correct the disease-causing mutated *KRT14* allele in EBS patient keratinocytes *in vitro*. Gene-corrected clones showed normal phenotype without characteristic mutant cytoplasmic aggregates in cell culture. DDEB is caused by dominant negative mutations in *COL7A1*, which codes for collagen 7 (C7) ^23, 24^. Shinkuma *et al.* ^25^ successfully used CRISPR-Cas9 to induce site-directed mutagenesis of the mutated *COL7A1* allele in iPSCs derived from DDEB patient keratinocytes. Edited cells expressed a truncated version of C7 that was incapable of forming deleterious trimers with WT C7 and would hypothetically allow for normal anchoring fibril formation at the DEJ ^25^.

Unlike EBS and DDEB, JEB and RDEB are inherited in an autosomal recessive manner. Therefore, CRISPR-Cas therapeutics for these disorders must achieve gene correction to allow for production of new, functional proteins. This can be accomplished through precise correction of the disruptive mutation (i.e., via CRISPR-Cas9 induced HDR with a gene-corrected donor template) or through methods that produce a functional protein without fully correcting the disease-causing mutation. For example, in specific cases, targeted deletions can be used to either remove a premature stop codon or causative mutation directly or to disrupt splicing signals, allowing for therapeutic exon skipping. JEB is caused by autosomal recessive mutations in genes encoding subunits of the heterotrimeric laminin-5 (laminin-332) protein (e.g., *LAMA3*, *LAMB3*, and *LAMC2*) ^26, 27^, and RDEB is caused by autosomal recessive mutations in *COL7A1* ^28, 29^. For both of these conditions, CRISPR-Cas9-mediated HDR has been used to successfully correct disease-causing mutations *ex vivo* in patient-derived primary keratinocytes ^30– 33^ and iPSCs ^34^. When grafted onto immunodeficient mice, gene-corrected keratinocytes displayed restored WT functionality and adhesion at the DEJ ^31– 33^. However, the efficiency of HDR during *ex vivo* gene modification remains low, particularly without antibiotic section protocols or other methods of enriching for modified cells. Exon skipping approaches have been explored for the treatment of RDEB. Exon 80 contains a common disease-causing point mutation in the *COL7A1* gene ^35^. By inducing targeted Cas9-mediated DSBs on either side of the mutation-bearing exon 80 in COL7A1, it is possible to mediate complete excision of the disease-causing mutation. Bonafant *et al.* delivered paired Cas9/sgRNA RNPs to patient keratinocytes *ex vivo* through electroporation. They were able to achieve efficient rates of deletion of exon 80, generating cells with restored C7 expression that, when grafted to immunodeficient mice, showed long-term dermal-epidermal adhesion ^36^.

Notably, Cas9-mediated therapies for RDEB have recently expanded to include *in vivo* approaches, with Wu and colleagues ^37^ restoring C7 function in an RDEB mouse model using the exon skipping approach. Wu *et al*. designed two sgRNAs targeting the 5’ and 3’ side of exon 80 and delivered them as an sgRNA/Cas9 ribonucleoprotein complex (RNP) via intradermal injection into mouse tail skin. They then directly electroporated the mouse tail to facilitate penetration of RNPs into epidermal stem cells. By causing DSBs on either side of the mutation-bearing exon 80 in COL7A1, they were able to achieve complete excision of the disease-causing mutation in epidermal stem cells. Electroporated mice displayed restored C7 function, and their dermal-epidermal adhesion area improved from 30% to 60% after one treatment ^37^. The success of this approach demonstrated the potential for CRISPR-Cas9-induced gene correction of epidermal stem cells *in vivo* without the cost and technical challenge of *ex vivo* cell modification. Still, however, several limitations of this method are apparent. Only 2% of epidermal cells were capable of being targeted with this novel *in vivo* delivery method, and no long-term follow-up to assess sustainability of the treatment could be performed. Moreover, potential off-target effects of the Cas9/sgRNA RNPs at sites other than exon 80 were not analyzed. Whether transdermal delivery of Cas9/sgRNA RNPs via electroporation would be safe and effective in humans has yet to be explored. However, it is likely that considerable pain and collateral damage may be associated with transdermal electroporation techniques ^38^, particularly for EB patients. Electroporation protocols for EB patients would require high voltages to penetrate the nuclei of epidermal stem cells and would need to be administered over large surface areas of skin.

***Epidermolytic palmoplantar keratoderma (EPPK)***. EPPK is an autosomal dominant keratin disease of the hereditary palmoplantar keratoderma (PPK) group characterized by an abnormal thickening of skin on the palms and soles ^39^. The disease is caused by dominant-negative missense mutations in the *KRT9* gene, leading to the formation of a mutant keratin 9 (K9) protein that interferes with the function of the wild-type K9 ^40^. EPPK has relatively localized disease manifestations that are well suited for targeted delivery of CRISPR therapeutics.

Luan and colleagues showed that through local delivery of a lentiviral (LV) vector carrying an sgRNA and Cas9 directed to the mutant *KRT9* allele, they could disrupt the formation of the mutant K9 protein *in vivo* in an EPPK mouse model and induce phenotypic correction of the disease ^40^. Mice who received nine subcutaneous injections of the Cas9/sgRNA-containing LV particles into their right forepaw over the course of 24 days displayed restored epidermal proliferation of the forepaw and a reduction in disease-associated K9 expression. Off-target effects of the genome editing system were reportedly minimal, but analyses were limited to 10 predicted off-target sites in the mouse genome ^40^. In addition, long-term effects of the treatment on the mice were not recorded, and there was no analysis of potential immune response to the LV Cas9 vector—an occurrence which has been reported for other LV vector systems ^41^. Still, this study demonstrates the potential for robust *in vivo* effects of CRISPR-Cas9-mediated gene editing, particularly for diseases that have localized manifestations.

### Cutaneous viruses

CRISPR-Cas systems, which evolved in bacteria to fight invading bacteriophages, have also been repurposed to serve a similar function in virally infected human cells. In human cells, CRISPR-Cas enzymes have the potential to target latent viruses that are able to escape eradication by immune surveillance and standard antiviral therapies. As such, there has been extensive research into the ability of CRISPR-Cas systems to target specific viral genomic sequences, enabling targeted disruption and even complete excision of components of the viral genome ^42^. In addition, researchers have leveraged the high sensitivity of certain Cas enzymes for the purposes of viral pathogen detection in human tissue samples ^43^. Cas12 and Cas13—two Cas enzymes that demonstrate indiscriminate trans-cleavage of ssDNA when activated by their guide-complementary target nucleotide sequence—enable ultrasensitive nucleic acid detection in viral biosensing systems such as DETECTR ^44^, SHERLOCK/SHERLOCKv2 ^45^ and HOLMES/HOLMESv2 ^46, 47^

Though most CRISPR-mediated antiviral research to date has focused on systemic viruses that lack primary cutaneous involvement, the unique accessibility of the skin suggests that CRISPR therapeutics and diagnostics might be more effectively applied to cutaneous viruses. Current research suggests CRISPR-Cas technology may be effective in detecting and modifying human papillomavirus (HPV), herpes simplex virus (HSV), and Kaposi sarcoma-associated herpesvirus (KSHV).

***HPV***. HPV is a dsDNA virus that infects the basal cells of stratified epithelium. Here, the virus has the potential to integrate its viral DNA into the host genome. The HPV E6 and E7 proteins of certain high-risk strains, including 16, 18, 31, 33, cause malignant transformation of epithelial cells by inactivating p53 and Rb, respectively ^48, 49^, and can lead to anogenital squamous cancers and other neoplasms. E7 expression from lower risk strains of HPV, particularly strains 6 and 11, is associated with the uncontrolled proliferation of epithelial cells that cause genital warts ^50, 51^.

Several researchers have effectively used CRISPR-Cas9 to disrupt of E6 and E7 genes in cervical cancer cells, both *in vitro* and *in vivo* in animal models ^47– 49, 52– 54^. One of the first antiviral CRISPR-Cas9 clinical trial in humans will involve in *vivo* targeting of E6/E7 in HPV-infected neoplastic cervical cells ^55^.

Though fewer studies have been performed with the aim of using CRISPR-Cas to cure the dermatological manifestations of HPV, researchers have begun to develop CRISPR-Cas constructs targeting the virus in HPV-associated anal cancer and genital warts. Hsu *et al.* ^56^ were able to successfully decrease tumor burden in a mouse model of HPV-16-associated anal cancer. Using an adeno-associated virus (AAV) vector, they delivered Cas9 nuclease in conjunction with two gRNAs: one gRNA specific for HPV-16 E6 and the other specific for HPV-16 E7. When delivered via three intratumoral injections over one week to mice with patient-derived xenografts of HPV-16 anal cancer cells, the CRISPR-Cas9 and dual-gRNA caused a twofold decrease in tumor volume, providing proof of concept for a novel *in vivo* gene editing strategy for HPV-16-associated anal cancer. For HPV-associated genital warts, CRISPR-Cas9 has been used to successfully target the E7 gene *in vitro*, promoting apoptosis of HPV-6 and -11-infected keratinocyte cell lines ^57^. Notably, however, the Cas9 and gRNAs were transfected transiently into the keratinocyte cell line and achieved only incomplete E7 deactivation. In addition, efficacy of the genome editing construct was not confirmed *in vivo*.

CRISPR-Cas systems hold potential for not only HPV therapeutics, but also HPV diagnostics. DETECTR is a nucleic acid biosensing system that uses the enzyme Cas12a and a ssDNA fluorescent reporting scheme to identify specific sequences in amplified dsDNA from human samples ^44^. Chen *et al.* ^44^ showed that by using their DETECTR platform, they could detect human papillomavirus (HPV) DNA in patient anal swab samples with attomolar sensitivity, even reliably distinguishing between different genotypes of the virus. Moreover, their assay was performed in just one hour and involved only isothermal amplification of DNA, suggesting that DETECTR could serve as a rapid, low-cost, point-of-care detection assay for HPV with similar sensitivity and specificity to conventional diagnostic PCR.

***Herpesviruses***. The herpesviruses are large dsDNA viruses that establish lifelong infection in human hosts ^58, 59^. HSV-1 and HSV-2 classically infect the oral and genital mucosal epithelium, respectively, leading to the local production of ulcers. After undergoing partial clearance by the host immune system, HSV establishes latent infection in the form of episomal DNA in sensory ganglia. KSHV, or human herpesvirus 8, infects human endothelial cells and is the causative agent of Kaposi’s sarcoma ^60^. Like HSV and other herpesviruses, KSHV remains latent in most infected cells. Latent herpes infections avoid immunological surveillance by limiting viral gene transcription ^61^ and are extremely difficult to treat.

As conventional therapies targeting viral replication are ineffective against latent virus, CRISPR-mediated targeting of viral DNA has emerged as an alternative approach for HSV, KSHV, and other herpesviruses. Roehm and colleagues ^62^ were able to completely abrogate HSV-1 replication in human fibroblast cells *in vitro* by disrupting two essential viral genes using CRISPR-Cas9. Other researchers have achieved similar success against HSV-1, inhibiting viral replication *in vitro* in oligodendroglioma cells and epithelial cells using Cas9/gRNA editing complexes without evident off-target effects ^62, 63^. For KSHV, researchers have demonstrated an ability to reduce the burden of KSHV in latently infected epithelial and endothelial cell lines by AAV-CRISPR-Cas9-mediated disruption of the KSHV latency-associated nuclear antigen (LANA) ^64^.

Still, challenges remain in the use of CRISPR-Cas for herpesviruses. First, despite demonstrated ability to abrogate active viral replication of HSV-1, it remains to be seen whether CRISPR-Cas can be effectively used to eradicate latent HSV-1 in neurons ^65^. Oh *et al.* were able to disrupt quiescent HSV-1 genomes, but at a much lower rate compared to HSV-1 virions in the lytic cycle ^66^. Because other targeted nucleases, such as MNs, have been shown to successfully target latent HSV-1 infection ^67^, it is suspected that current failure to eliminate latent HSV-1 using CRISPR-Cas9 may be due to epigenetic modifications of the latent HSV-1 genome that block Cas9 activity ^65, 68^. Moreover, further demonstration of the effectiveness of anti-HSV and anti-KSHV CRISPR-Cas systems *in vivo* will also be important. For HSV-1 and -2, *in vivo* delivery may be simpler than for other systems because latent HSV-1 and 2 have very specific tropism for the trigeminal and sacral ganglia, respectively, and possible delivery strategies could be designed to specifically home to these ganglia.

### Cutaneous bacterial infections

In addition to viral infections, CRISPR-Cas technology also shows promise against resistant bacterial infections ^69, 70^. Bacterial infections that are resistant to common antibiotics represent a growing public health concern ^71, 72^. Yet, despite this risk, antibiotics continue to be overprescribed ^73^ while the development of novel antimicrobials for new pathogens lags behind ^74^. Recently, CRISPR-Cas antimicrobials have emerged as a novel treatment strategy against bacterial infections ^69, 70^. Specifically, CRISPR-Cas9 has been leveraged to selectively remove antimicrobial resistance genes from populations of bacteria, re-sensitizing populations of bacteria to common antimicrobials ^75, 76^. Though systemic delivery of CRISPR-based antimicrobials remains a challenge, the accessibility of the skin enables the delivery of CRISPR constructs via convenient topical formulations ^69^ and places cutaneous bacterial infections at the forefront of CRISPR antimicrobial research.

***Staphylococcus aureus***. *Staphylococcus aureus*, a common cutaneous bacterial pathogen known for its antimicrobial resistance, is responsible for 76% of all skin and soft tissue infections ^77^ and is associated with high morbidity and mortality ^78^. Antimicrobial resistance to *S. aureus* continues to emerge as the pathogen gains plasmids and other mobile genetic elements that confer antibiotic resistance and virulence genes ^79^. Moreover, outbreaks remain common as the pathogen maintains a high prevalence in the population, asymptomatically colonizing the nostrils of 20–30% of healthy adults ^80^.

Bikard and colleagues ^75^ developed a novel approach to target virulent strains of *S. Aureus* using CRISPR-Cas9. They developed gRNAs targeted to specific *S. Aureus* antimicrobial resistance genes, including the methicillin resistance gene, *mecA*. When delivered with Cas9 via a phage capsid to mixed populations of bacteria *in vitro*, these gRNA/Cas9 constructs were able to eradicate resistant *S. Aureus* strains and completely remove specific plasmids carrying antimicrobial resistance genes. Moreover, when delivered topically to a mouse model of *S. Aureus* skin colonization *in vivo*, these gRNA/Cas9 constructs were able to significantly decrease colonization by resistant bacteria. These results demonstrated promise for the topical application of CRISPR antimicrobials *in vivo* and laid the foundation for future multiplexed CRISPR antimicrobials designed to simultaneously target either several bacterial species or multiple gene sequences within the same bacterium. These results also demonstrated the potential for CRISPR antimicrobials to modulate the cutaneous microbiome. The role of the cutaneous microbiome in dermatologic disease continues to unfold as researchers analyze metagenomic sequencing data from varied skin samples ^81^. For example, reduced microbiome diversity and increased S. aureus skin colonization has been implicated in the pathogenesis of atopic dermatitis ^82^. The potential role for CRISPR antimicrobials in atopic dermatitis patients has yet to be explored, but antimicrobials targeting pathogenic S. aureus strains may complement strategies designed to augment commensal bacterial populations in the cutaneous microbiome ^83^.

### Melanoma

Some of the first CRISPR-Cas clinical trials in humans have involved the use of CRISPR-Cas technology in immunotherapy for cancers, including melanoma and non-small-cell lung cancer (NSCLC) ^4^. Much of this research has centered on the use of gene editing to inactivate key immune checkpoint inhibitors such as programmed cell death protein-1 (PD-1) and cytotoxic T-lymphocyte-associated protein-4 (CTLA-4)—two proteins that normally inhibit the anti-tumor cytotoxic effect of endogenous and exogenous T cells ^84^.

Melanoma is well-known to have exceptional immunogenic potential, owing largely to the high mutational burden that drives the formation of immune-stimulating neoantigens ^85^. Thus, under optimal conditions, melanoma cells are particularly susceptible to destruction by the human immune system ^86^. Yet, clinically, the tumor microenvironment in melanoma is highly immunosuppressive ^87^, and advanced disease has exceptionally poor treatment response ^88^. Consequently, melanoma serves as an ideal target for immunotherapies that are designed to relieve tumor immunosuppression.

The first human trial designed to test the use of CRISPR-Cas for melanoma builds on the demonstrated success of previous immunotherapies, including PD-1 inhibitors ^89^ and T cells transduced with the NY-ESO-1 T-cell receptor (TCR) ^90^. Specifically, researchers aim to amplify the therapeutic effects of these existing approaches by using CRISPR-Cas9 to knock out PD-1 gene loci in autologous NY-ESO-1 TCR-transduced T cells. Autologous T cells are first taken from a patient and transduced with a LV vector that expresses the NY-ESO-1 TCR, priming them to recognize a highly immunogenic NY-ESO-1 antigen expressed on melanoma cells ^91^. They are then electroporated with RNA-guided CRISPR-Cas9 nucleases designed to disrupt the expression of both PD-1 and the endogenous TCR subunits, TCRα and TCRβ ^4^. Disrupting PD-1 prevents immune suppressive signaling, and blocking the endogenous TCR subunits inhibits aberrant immune responses that may result from TCR-mediated targeting of unknown antigens. With re-introduction of these melanoma-targeted, immune-avid T cells, researchers hope to obtain a more robust tumor-specific immune response—a response that has been difficult to achieve with previous T-cell therapies for solid tumors ^92^. Moreover, beyond having an enhanced anti-tumor effect, such an approach is also expected to minimize unwanted treatment side effects. Using this treatment model, PD-1 blockade will be limited to the specific TCR-transduced T-cells that are designed to home to and attack NY-ESO-1-expressing melanoma cells. This stands in contrast to traditional anti-PD1 receptor antibodies which, when administered systemically, have the potential to affect T-cells more broadly and cause widespread immunogenic side effects ^93^.

## Perspectives and future directions

Taken together, a wide variety of studies underscore the potential for CRISPR-based therapeutics for genodermatoses, cutaneous infections, and melanoma. Future research will likely continue to expand on this success, with the aim of increasing the translatability of CRISPR therapeutics as well as developing expanded strategies to target other dermatologic diseases.

There are many other genetic skin disorders and cutaneous infections that could be targeted with CRISPR-Cas therapeutics. Pachyonychia congenita and xeroderma pigmentosum have been targeted with RNAi-based therapies ^94^ and designer nucleases ^95^, respectively, and could likely also be targeted with CRIPSR-based genome engineering. Rare cases of EB are caused by multiple co-occurring mutations ^96– 99^ and could be treated with unique CRISPR-Cas constructs targeting several genetic loci ^4^. Moreover, with the advent of hypoimmunogenic universal donor iPSCs—iPSCs that are genetically modified by CRISPR-Cas9 to avoid inciting a host immune response ^100, 101^—existing *ex vivo* gene modification strategies for genodermatoses could be applied to patients more broadly. Despite concern for the tumorigenic potential of iPSCs ^102^, CRISPR-edited iPSCs were not associated with tumorigenesis in a mouse model of RDEB32. By optimizing the re-differentiation process of iPSCs and excluding cells with oncogenic potential, iPSCs may prove safe for clinical application ^103^. 

There are many cutaneous viruses that hold promise for targeting with gene editing technologies, including the oncoviruses, Merkel-cell polyomavirus (MCPyV), and human T-cell leukemia virus type 1 (HTLV-1). MCPyV causes up to 80% of Merkel cell carcinomas (MCC) and is randomly integrated into different sites in the MCC tumor genome ^104^. Excision of MCV from MCC tumor cells could represent a potential therapeutic strategy for aggressive MCCs that are often refractory to many current therapies ^104– 106^. Preliminary studies in Merkel cell cancer cell lines demonstrate that CRISPR-Cas9-mediated disruption of the MCPyV tumor antigens leads to diminished cell growth ^107^. HTLV-1, the retrovirus that causes HTLV-1-associated/tropical spastic myelopathy and adult T cell leukemia/lymphoma, has yet to be explored as a target for CRISPR-Cas therapeutics. Studies focusing on HIV ^108– 111^—a similarly structured retrovirus—would suggest that HTLV-1 could likely be completely excised from infected cells using CRISPR-Cas9. The relative genetic conservation of HTLV-1 relative to HIV-1 ^112^ would make it an even more appropriate candidate for targeting by RNA-guided endonucleases.

As engineered T cell therapies continue to expand, clinical trials will begin to test the use of increasingly safe and effective CRISPR-edited TCR-transduced and chimeric antigen receptor (CAR) T cells for melanoma. CRISPR gene editing technology will facilitate the creation of universal donor T cells with disrupted endogenous TCR and human leukocyte antigens class I (HLA-Is) ^113^, allowing T cells extracted from healthy donors to be used for the treatment of patients with any HLA type. In addition, T cell therapies will become safer and more controllable through the addition of inducible safety genes to genetically engineered T cells. For example, cells engineered with an inducible caspase-9 gene will undergo apoptosis in the presence of the small molecule inducer AP1903 ^114^. This “kill switch” allows for drug- or small-molecule-induced inactivation of edited cells in the case of an adverse reaction.

Lastly, CRISPR-Cas technology will likely develop clinical utility in the diagnosis of dermatological disease. CRISPR-Cas diagnostic platforms using Cas9 ^115^, Cas12 ^44, 47, 116^, Cas13 ^45^, and Cas14 ^117^ have the potential to revolutionize the detection of nucleic acid sequences, allowing for the ultrasensitive, low-cost, and portable detection of cutaneous viruses and single point mutations in cutaneous tumors ^117, 118^.

## Challenges to therapeutic application

There remain several challenges to the widespread application of CRISPR-based therapeutics. The studies reported here largely demonstrate the ability of CRISPR-Cas systems to treat human disease both in cell culture models and through *ex vivo* modification of primary patient cell lines. While such methods currently represent the safest approach to gene editing in humans, such a technique is technologically challenging and of limited use for routine clinical practice. *In vivo* treatment options would be ideal, but fewer studies have explored these possibilities. Studies on the *in vivo* treatment of non-dermatologic disorders, including Duchenne muscular dystrophy ^119– 121^, hereditary liver disease ^122– 124^, congenital eye disease ^125^, and Huntington Disease ^126^ have shown tremendous promise in animal models. However, of the studies in dermatology focused on *in vivo* delivery of CRISPR-therapeutics, all have been limited to localized effects in mouse models, and none have demonstrated high efficiency or success in long-term follow up ^37, 40, 56^.

For *in vivo* genome editing via CRISPR-Cas technology to be clinically translatable not only in dermatology but also in other fields, there are several major challenges that are yet to be effectively addressed. CRISPR-Cas guide RNAs and nucleases must 1) be optimized for robust and robust and specific on-target effects with minimal off-target effects, 2) be delivered efficiently to specific human cells, and 3) have minimal antigenic properties so that they are accepted by human immune systems ^127^. Novel CRISPR-Cas enzymes and delivery systems are being developed to tackle these challenges. To improve specificity of CRISPR-Cas9, researchers have modified Cas9 construction ^128^, optimized sgRNA design ^129^, and developed a CRISPR-Cas9 double nickase approach ^130^ that introduces only single-strand nicks at target sites. Newly developed Cas9 variants (enhanced specificity Cas9 [eSpCas9] ^131^, high-fidelity Cas9 [Cas9-HF1] ^128^, hyper-accurate Cas9 [HypaCas9] ^132^, evoCas9 ^133^, and xCas9 ^134^) allow for improved gene editing specificity. HDR efficiency is augmented by chemical NHEJ inhibitors ^8^, modified HDR templates, and polymer-stabilized Cas9 RNPs ^135^. In addition, researchers have developed sensitive methods to scan the entire genome for unintended off-target genome editing effects, including GUIDE-Seq ^136^, Digenome-seq ^137^, SITE-seq ^138^, CIRCLE-Seq ^139^, and, most recently, VIVO ^140^. Such methods would allow for screening of off-target effects of a given Cas/gRNA construct prior to therapeutic use.

Novel delivery methods are also being explored that expand upon traditional AAV- and LV-associated delivery systems. Gene delivery methods with viral vectors have the potential to cause integrational mutagenesis and lifelong Cas9 expression in cells. For this reason, delivery of Cas9 nuclease by way of a transiently expressed Cas9/sgRNA RNP may be preferred. Scientists have developed lipid nanoparticles that can carry CRISPR-Cas nucleotide sequences or RNP complexes that are targeted to specific organs ^141– 143^. Still, it will be challenging to achieve systemic delivery of such therapies, particularly for bloodborne illnesses that would require the delivery of CRISPR-Cas constructs to every circulating B or T cell ^42^.

Lastly, to limit the possible immunologic response to Cas9 by preformed antibodies in human serum ^144^, researchers have discovered novel Cas enzymes such as the structurally distinct Cas12e and Cas12d from ground bacteria ^145^. CRISPR therapeutics employing nucleases from bacteria to which humans are not exposed may not be subject to pre-existing immunity, allowing for a more robust genome editing effect.

## Conclusion

Though significant work remains to be done prior to its widespread therapeutic use in humans, preliminary research suggests great potential for CRISPR-Cas technology in the treatment of dermatological pathologies. As researchers continue to optimize delivery methods and off-target screening approaches, more human trials for the treatment of dermatologic diseases with CRISPR-based gene editing therapeutics will likely be initiated.

## Data availability

No data are associated with this study.
