# Supplementary material for: Gene editing in dermatology: Harnessing CRISPR for the treatment of cutaneous disease
Source: F1000Res. 2020 Oct 7;9:281. Originally published 2020 Apr 23. [Version 2] doi: 10.12688/f1000research.23185.2 (PMC7265575; doi:10.12688/f1000research.23185.2)
Supplement: Peer review history

# Peer review: Reviewer response for version 2

**DOI:** 10.5256/f1000research.29935.r72624

**Published:** 2020-10-19

**Authors:** Jillian Richmond

**Affiliations:** 1 Department of Dermatology, University of Massachusetts Medical School, Worcester, MA, USA

Thank you for addressing my comments.

Is the review written in accessible language?

Yes

Are all factual statements correct and adequately supported by citations?

Yes

Are the conclusions drawn appropriate in the context of the current research literature?

Yes

Is the topic of the review discussed comprehensively in the context of the current literature?

Yes

Reviewer Expertise:

dermatology research, autoimmunity, skin diseases

I confirm that I have read this submission and believe that I have an appropriate level of expertise to confirm that it is of an acceptable scientific standard.

# Peer review: Reviewer response for version 1

**DOI:** 10.5256/f1000research.25593.r63774

**Published:** 2020-06-01

**Authors:** Jillian Richmond

**Affiliations:** 1 Department of Dermatology, University of Massachusetts Medical School, Worcester, MA, USA

In this review, Baker & Hayden describe the field of gene editing for the treatment of dermatologic conditions. This is a very well-written and interesting review. I only have minor suggestions prior to publication.

**Minor:**

- In your discussion of NHEJ and HDR, could you provide specifics about the efficiency of HDR (as a freq, etc)?
- Under "Genodermatoses" you have a few sentences that seem redundant, please consider streamlining your intro paragraph before going into specific disease areas.
- Last paragraph under EB: any comment/concern about electroporating a mouse tail? Feasibility in humans? Side effects?
- In the melanoma section, any thoughts about "kill switch" T cells?
- Maybe include a brief discussion about the skin microbiome, either in the bacteria section or future directions section? Could focus on the impact of gene editing on microbiome dynamics, or therapeutic potential for correcting dysbioses.

Is the review written in accessible language?

Yes

Are all factual statements correct and adequately supported by citations?

Yes

Are the conclusions drawn appropriate in the context of the current research literature?

Yes

Is the topic of the review discussed comprehensively in the context of the current literature?

Yes

Reviewer Expertise:

dermatology research, autoimmunity, skin diseases

I confirm that I have read this submission and believe that I have an appropriate level of expertise to confirm that it is of an acceptable scientific standard.

# Response

**Published:** 2020-09-27

**Authors:** Catherine Baker

**Affiliations:** Geisel School of Medicine at Dartmouth, USA

Dear Dr. Richmond,

 Thank you for your thoughtful review of our manuscript. We have carefully reviewed the comments and have revised the manuscript accordingly. Our responses are given in a point-by-point manner below.

  

1. In your discussion of NHEJ and HDR, could you provide specifics about the efficiency of HDR (as a freq, etc)?
  1. This is a difficult question because relative efficiencies of NHEJ and HDR vary quite widely depending on cell type and conditions. A sentence addressing this issue, as well as two references to articles addressing this topic, were added to the HDR/NHEJ discussion in “Mechanisms of genome engineering with CRISPR-Cas.”
2. Under "Genodermatoses" you have a few sentences that seem redundant, please consider streamlining your intro paragraph before going into specific disease areas.
  1. Repetitive words/sentences were eliminated in this paragraph.
3. Last paragraph under EB: any comment/concern about electroporating a mouse tail? Feasibility in humans? Side effects?
  1. Two sentences on the feasibility of electroporation for human patients with EB were added.
4. In the melanoma section, any thoughts about "kill switch" T cells?
  1. A paragraph on engineered T-cell therapies, including “kill switch” T cells, was added to the “Perspectives and future directions” section.
5. Maybe include a brief discussion about the skin microbiome, either in the bacteria section or future directions section? Could focus on the impact of gene editing on microbiome dynamics, or therapeutic potential for correcting dysbioses.
  1. In “Cutaneous bacterial infections,” a section on the potential role for CRISPR in modulating the cutaneous microbiome was added, particularly as it pertains to atopic dermatitis and S. aureus colonization.

# Peer review: Reviewer response for version 1

**DOI:** 10.5256/f1000research.25593.r62746

**Published:** 2020-05-18

**Authors:** Bindu Hegde

**Affiliations:** 1 Department of Neurological Surgery, University of California, San Francisco, San Francisco, CA, USA

In this review article, Baker and Hayden discuss the role of CRISPR technology in genome editing for dermatological diseases. Overall, the manuscript is well-written with clear and unambiguous language. The article highlights the studies that use CRISPR-Cas 9 approach for gene editing in various cutaneous diseases and provides a balanced assessment by noting both advantages and limitations of using this technology. However, it could improved with the following suggestions:

*Page 4 - *Since EB can be caused by mutation in several genes and multiple mutations can be observed within the same patient, strategies correcting a single mutation may not reverse the disease completely. How would some of these studies, which correct a single mutation, translate clinically? Are there any efforts to correct multiple mutations at once using CRISPR?  

*Page 7 -* iPSCs are an attractive tool for cell therapy in some ways because of their low immunogenicity. However, there are several safety concerns with these cells. They could acquire mutations while being expanded in culture and develop into teratomas (Dinella 2014 ^1^). What methods are used to overcome these concerns?

*Page 7 - *Challenges of therapeutic application should be discussed in more detail. Low efficiency of gene editing is also one of the drawbacks. What are the new techniques being applied to overcome this? (For example, one of the ways to improve the efficiency of HDR is the use of NHEJ inhibitors)

*Page 7* - In your discussion of strategies to improve the specificity of Cas9, include the possibility of using several modifications of Cas9 like high-fidelity Cas9 (eSpCas9), evolved Cas9 (evoCas9) in addition to the ones you have mentioned.

Is the review written in accessible language?

Yes

Are all factual statements correct and adequately supported by citations?

Yes

Are the conclusions drawn appropriate in the context of the current research literature?

Yes

Is the topic of the review discussed comprehensively in the context of the current literature?

Partly

Reviewer Expertise:

Drug discovery, Immunology, Cancer immunotherapy

I confirm that I have read this submission and believe that I have an appropriate level of expertise to confirm that it is of an acceptable scientific standard.

## References

[1] : Use of Induced Pluripotent Stem Cells in Dermatological Research. *Journal of Investigative Dermatology*.2014;134(8) : 10.1038/jid.2014.238 1-5 10.1038/jid.2014.238 PMC452753225029325

# Response

**Published:** 2020-09-27

**Authors:** Catherine Baker

**Affiliations:** Geisel School of Medicine at Dartmouth, USA

Dear Dr. Hegde,

 Thank you for your thoughtful review of our manuscript. We have carefully reviewed the comments and have revised the manuscript accordingly. Our responses are given in a point-by-point manner below.

 

1. Since EB can be caused by mutation in several genes and multiple mutations can be observed within the same patient, strategies correcting a single mutation may not reverse the disease completely. How would some of these studies, which correct a single mutation, translate clinically? Are there any efforts to correct multiple mutations at once using CRISPR? 
  1. A sentence regarding the potential use of CRISPR for targeting rare cases of EB caused by >1 mutation was added to the “Perspectives and future directions” section.
2. iPSCs are an attractive tool for cell therapy in some ways because of their low immunogenicity. However, there are several safety concerns with these cells. They could acquire mutations while being expanded in culture and develop into teratomas (Dinella 2014). What methods are used to overcome these concerns?
  1. The challenges of gene editing with iPSCs are alluded to in the last paragraph of the “Mechanisms of genome engineering with CRISPR-Cas” section.
3. Challenges of therapeutic application should be discussed in more detail. Low efficiency of gene editing is also one of the drawbacks. What are the new techniques being applied to overcome this? (For example, one of the ways to improve the efficiency of HDR is the use of NHEJ inhibitors)
  1. Expanded second paragraph of “Challenges to therapeutic application” to include mention of NHEJ inhibitors and brief mention of other strategies to improve efficiency of Cas9-mediated HDR.
4. In your discussion of strategies to improve the specificity of Cas9, include the possibility of using several modifications of Cas9 like high-fidelity Cas9 (eSpCas9), evolved Cas9 (evoCas9) in addition to the ones you have mentioned.
  1. Cas9 variants with enhanced specificity have been listed as strategies to improve the specificity of Cas9 (second paragraph of “Challenges to therapeutic application” section).
